# Organizational Heterogeneity of Vertebrate Genomes

**DOI:** 10.1371/journal.pone.0032076

**Published:** 2012-02-27

**Authors:** Svetlana Frenkel, Valery Kirzhner, Abraham Korol

**Affiliations:** Department of Evolutionary and Environmental Biology and Institute of Evolution, University of Haifa, Mount Carmel, Haifa, Israel; Ecole Normale Supérieure de Lyon, France

## Abstract

Genomes of higher eukaryotes are mosaics of segments with various structural, functional, and evolutionary properties. The availability of whole-genome sequences allows the investigation of their structure as “texts” using different statistical and computational methods. One such method, referred to as Compositional Spectra (CS) analysis, is based on scoring the occurrences of fixed-length oligonucleotides (k-mers) in the target DNA sequence. CS analysis allows generating species- or region-specific characteristics of the genome, regardless of their length and the presence of coding DNA. In this study, we consider the heterogeneity of vertebrate genomes as a joint effect of regional variation in sequence organization superimposed on the differences in nucleotide composition. We estimated compositional and organizational heterogeneity of genome and chromosome sequences separately and found that both heterogeneity types vary widely among genomes as well as among chromosomes in all investigated taxonomic groups. The high correspondence of heterogeneity scores obtained on three genome fractions, coding, repetitive, and the remaining part of the noncoding DNA (the genome dark matter - GDM) allows the assumption that CS-heterogeneity may have functional relevance to genome regulation. Of special interest for such interpretation is the fact that natural GDM sequences display the highest deviation from the corresponding reshuffled sequences.

## Introduction

Unraveling the structural organization of complex eukaryotic genomes is one of the most important problems in current genomics. A plentiful amount of genomes has been sequenced and is available for further analysis. Long DNA sequences, like chromosomes or entire genomes, are known to be heterogeneous in their structural aspects, such as GC content (isochores), CpG distribution, copy number variation, repetitive DNA content, and distribution of indels. Furthermore, they are heterogeneous in their functional and evolution-related features, including dynamics of DNA replication, protein and non-protein-coding DNA content, codon usage, level and tissue-specificity of gene expression, distribution of conserved and ultra-conserved regions, recombination and mutation hot and cold spots, and SNPs and LD-blocks [1]–[6]. Comparative analysis of mutation rates in mammals indicates that parallel syntenic blocks, rather than entire chromosomes, may represent the units of intragenomic heterogeneity of mutation rates [7]. However, until recently, most of the genome analyses were devoted to the coding space; hence, a major part of sequence organization of eukaryotes remained poorly studied, including intragenomic heterogeneity. One of the main exclusions was, and still remains, intragenomic variation in GC content.

### GC content and CpG islands

A simple measure of compositional organization of nucleotide sequences is the molar ratio of G+C in DNA, or GC content. GC content displays wide variation within genomes, chromosomes, and chromosome segments [8]–[10]. Long homogeneous regions with certain GC content are called GC isochores [8]; the resolution of isochore maps of the human genome is higher than the resolution of classical Giemsa and Reverse bands [11]. GC content is known to be strongly correlated with biological features of genome organization, such as dynamics of DNA replication [12], [13], gene density [14], [15], level and tissue-specificity of transcription [16], mutation and recombination rates [17], [18]. GC content in non-mammalian vertebrate genomes is less variable than in mammals, but GC-based segmentation of these genomes is still possible [19]. However, as shown by Nekrutenko and Li [20] and supported by our results, heterogeneity of eukaryotic genomes is not confined to the variation of GC isochores. Li [10] showed that some isochores are heterogeneous in their GC content and Costantini and Bernardi [21] found different di- and tri-nucleotide patterns in diverse isochores. Therefore, GC-based characteristics are not sufficient for comprehensive investigations of eukaryotic genome heterogeneity and its relevance to functions and the evolution of genomes. Well known since 1987 [22], CpG islands were found to be associated with gene abundance [23], gene expression [16], local abundance of Alu-sequences and (obviously) with GC content of the region [24]. GC content and CpG islands play a role in tissue-specific differentiation and cancer development [25], [26].

### Oligonucleotide-based methods

A group of methods for analyzing genome organization based on counting oligonucleotide “word” (or k-mer) occurrences was proposed in the 1980s [27]–[30]. The main purpose of such alignment-free analysis is the determination of genomic characteristics (signatures, patterns). It allows differentiation of regions within a genome, comparison of genomes of diverse species, and many other applications [31], [32]. Genome-specific characteristics are employed in phylogenetic analyses [33] or in species recognition using their relatively short DNA fragments as training inputs for classification algorithms [34], [35]. Region-specific characteristics can be used for the detection of certain elements in DNA sequences such as candidate regulatory elements [36]–[38], promoter regions [39], and repetitive elements that were not found before [40]. This method proved useful for the detection and determination of the origin of alien DNA segments in studies of horizontal gene transfer [41]–[43] and duplications of genomic segments [44]. In addition, the oligonucleotide-counting methods are used for preliminary searches of candidates for subsequent gene alignment [45] as well as whole-genome sequence comparisons [30], [46]–[49].

One of the word-counting methods, referred to as Compositional Spectra Analysis (CSA), based on scoring the occurrences of fixed-length words from a predefined set (“vocabulary”) with a chosen level of allowed mismatching, was suggested by Kirzhner et al. [50] and used for genome comparisons of different species. CSA allows generating species- or region-specific characteristics of the genome, regardless of their length and the presence of coding DNA. Here we employ CSA in the investigation of organizational heterogeneity of vertebrate genomes. In addition to the entire DNA sequence, we analyze its coding and two noncoding parts separately: repeated DNA and the rest of the sequence, which we refer to as genome dark matter (see also [51]–[54] for a similar use of this terminology).

Our results provide new evidence that genome structure is more complicated than just a combination of regions with diverse GC contents; this structure varies at the level of chromosomes, but is shared to a considerable extent between the coding space and the considered two parts of noncoding space. In our CS analysis, we investigated three heterogeneity types: compositional (i.e., variability of nucleotide abundances along the sequence), organizational (i.e., variability of nucleotide order along the sequence), and total CS heterogeneity, which is a result of a complicated interaction of the two former types.

### Employed sequences

Chromosomal complete and repeat-masked sequences of 19 vertebrate species and genomic complete and repeat-masked sequences (available as scaffolds) of 26 vertebrate species were downloaded from Ensembl (release 57, http://mar2010.archive.ensembl.org/index.html). We analyzed several taxonomic groups of species (Table 1), including five groups of mammals: apes (4) and other primates (5), ungulates (4), rodents (5) and “other mammals” group, consisting of 14 diverse species; and four groups of non-mammalian vertebrates: marsupials (2), birds (3), fishes (5) and “other vertebrates” (anole lizard, frog, and platypus).

**Table 1:** List of genomes used for analyses.

English name	Latin name	Ensembl assembly name	English name	Latin name	Ensembl assembly name
**Apes**			Hedgehog	*Erinaceus europaeus* *	HEDGEHOG
Gorilla	*Gorilla gorilla*	gorGor3	Cat	*Felis catus* *	CAT
Human	*Homo sapiens*	GRCh37	Elephant	*Loxodonta africana* *	loxAfr3
Chimpanzee	*Pan troglodytes*	CHIMP2.1	Microbat	*Myotis lucifugus* *	MICROBAT1
Orangutan	*Pongo pygmaeus*	PPYG2	Pika	*Ochotona princeps* *	PIKA
**Other primates**			Hyrax	*Procavia capensis* *	proCap1
Marmoset	*Callithrix jacchus* *	calJac3	Megabat	*Pteropus vampyrus* *	pteVam1
Macaque	*Macaca mulatta*	MMUL_1	Shrew	*Sorex araneus* *	COMMON_SHREW1
Mouse lemur	*Microcebus murinus* *	micMur1	Tree shrew	*Tupaia belangeri* *	TREESHREW
Bushbaby	*Otolemur garnettii* *	BUSHBABY1	Dolphin	*Tursiops truncatus* *	turTru1
Tarsier	*Tarsius syrichta* *	tarSyr1	**Marsupials**		
**Rodents**			Wallaby	*Macropus eugenii* *	Meug_1.0
Guinea pig	*Cavia porcellus* *	cavPor3	Opossum	*Monodelphis domestica*	BROADO5
Kangaroo rat	*Dipodomys ordii* *	dipOrd1	**Birds**		
Mouse	*Mus musculus*	NCBIM37	Chicken	*Gallus gallus*	WASHUC2
Rat	*Rattus norvegicus*	RGSC3.4	Turkey	*Meleagris gallopavo*	UMD2
Squirrel	*Spermophilus tridecemlineatus* *	SQUIRREL	Zebra finch	*Taeniopygia guttata*	taeGut3.2.4
**Ungulates**			**Fishes**		
Cow	*Bos taurus*	Btau_4.0	Stickleback	*Gasterosteus aculeatus* *	BROADS1
Horse	*Equus caballus*	EquCab2	Zebrafish	*Danio rerio*	Zv8
Pig	*Sus scrofa*	Sscrofa9	Medaka	*Oryzias latipes*	MEDAKA1
Alpaca	*Vicugna pacos* *	vicPac1	Fugu	*Takifugu rubripes* *	FUGU4
**Other mammals**			Tetraodon	*Tetraodon nigroviridis*	TETRAODON8
Dog	*Canis familiaris*	BROADD2	**Other vertebrates**		
Sloth	*Choloepus hoffmanni* *	choHof1	Anole lizard	*Anolis carolinensis* *	AnoCar1
Armadillo	*Dasypus novemcinctus* *	dasNov2	Platypus	*Ornithorhynchus anatinus*	OANA5
Lesser hedgehog tenrec	*Echinops telfairi* *	TENREC	Frog	*Xenopus tropicalis* *	JGI4.1

*incomplete genome sequences.

A human predicted gene list that includes descriptions and positions of genes and pseudogenes of protein-coding and RNA-coding sequences was imported from Ensembl BioMart Database (http://www.ensembl.org/biomart/martview).

## Methods

### Computation of Compositional Spectrum

Consider a set *W* (vocabulary) including *n* different words (oligonucleotides) *w*_i_ of length *L* from standard {G, C, A, T} alphabet. Obviously, what follows is *n*≤4*^L^*. By moving along a DNA sequence of length *K* one position per step with a window of length *L*, we can collect *M = K−L+1* subsequences of length *L*. Comparing of these subsequences with all words *w*_i_ from the set *W* with a certain number of allowed replacements (mismatches) *r* allows us to calculate the number of imperfect matches *m*_i_ = *m*(*w_i_*) of each word with the analyzed sequence. Thus, the appearance of a 5-mer CTATG in a sequence of 54 bp length CTTTGAGTGGCAATAGAGCATTTCAGTAATTGTACCTCTATCCCTACAAGGAAC with *r* = 2 is *m*(CTATG) = 6. This word would not be found in the sequence upon *r* = 0 (without mismatches), but with one mismatch it was found twice (CT**T**TG and CTAT**C**) and with two mismatches four times (C**A**AT**A**, CT**C**T**A**, CTA**CA**, and C**A**A**G**G).

The frequency distribution *F(W,S)* based on frequencies *f*_i_ = *m*_i_/*M* is referred to as Compositional Spectrum (CS) of sequence *S* relative to a set of words *W* [50]. In our analysis, sets of words were generated by using an algorithm that randomly chooses the first word of length *L* in a set, and then examines all *L*-mers in alphabetical order. Every word, which differs from all previously generated words by a chosen number of letters, is included in the set. When the algorithm reaches the last word in the alphabet order (TTTTTTTTTT), it begins from the first word (AAAAAAAAAA), finishing the search when the first word included in the set is reached. Number *n* of words in a set does not depend on the first word included. Since this set contains the words with all variations of letters abundances, it is unbiased in its sensitivity to different genomic sequence. However, one may prefer to use specific biased sets of words, e.g., overpopulated by GC- or AT. For *L* = 10 and mismatch *r*≤3, we employed sets of 256 words with differences between words by at least six positions, e.g., ATGCTGTCAT, ATGCATAGCA, ATGCCACTGC, ATGCGCGATG, etc. Thus, with *r* = 3 any 10-letter subsequence from the targeted sequence coincides with no more than one word from such set. However, some oligonucleotides in the targeted genome sequence may remain uncovered.

### Coverage of targeted sequences

The maximal number of imperfect matches *μ* for any word of length *L* in the alphabet with *A* letters and allowed the mismatching level *r* per word, can be calculated using the following formula:
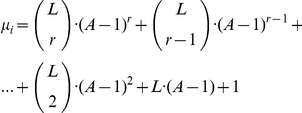
For *L* = 10, *r* = 3, the maximal number of matches for any word in the standard 4-letter alphabet is 3676, which gives us 941056 matches for a set of 256 words, i.e. 86.7% from all possible 10-letter words. We employed a few sets of words (obtained by the method described above) to analyze the human chromosome sequences using different variations of segment length and found that all sets cover 90–91% of chromosome sequences in all cases. This excess over the expected coverage is probably a result of the fact that the human genome does not use all possible 10-letter words.

### Justification of the selected parameter values

The sensitivity of CS to the sequence organizational features increases with the increasing of words length used. However, very long oligonucleotide words could be too specific with vast majority being presented with zero occurrences. The words of 8–15 letters long are most appropriate for our purposes [50]. Increasing the allowed number of replacements lowers the sensitivity of CS to sequence heterogeneity while decreasing *r* reduces the number of matches *μ* and leads to lower coverage of the targeted sequence. To avoid this effect one could use a richer set of words (e.g., all 1,048,576 10-mers at *r* = 0 giving 100% coverage without mismatching at all). However, as noted above, decreasing *r* reduces the observed frequencies of words and leads to a huge prevalence of zero frequencies in CS of short-to-moderate sequences (∼100–1000 kb), thereby complicating sequence comparisons. Longer sequences (≫1 Mb) would relax this problem, but the resolution of such an analysis is not sufficient for investigation of sequence heterogeneity. The segment size of 100 kb length seems to be most suitable for our goals; moreover, this length is comparable with common GC isochore lengths.

### Calculating CS-distances between DNA sequences

Let us define a measure of the differences between two sequences, *S*_1_ and *S*_2_, as the CS-distance *d* between their spectra *F(W*, *S*_1_) and *F(W*, *S*_2_). For this purpose, we use a metric based on the Spearman rank correlation coefficient [50]. Namely, we calculate Spearman rank correlation coefficient *r*_s_ between two CS corresponding to the compared sequences:
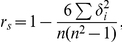
where *δ_i_* is the difference between the ranks of the *i* ^th^ word frequency in the compared spectra, and *n* is the number of words in the set. The obtained correlation coefficient *r*_s_ is used to determine CS distance *d* between the compared sequences *S*_1_ and *S*_2_:
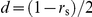
The maximal value *d* = 1 corresponds to strictly reverse compositional spectra (i.e., *r*_s_ = −1), whereas minimal value *d* = 0 corresponds to identical spectra (i.e., *r*_s_ = 1). However, this method does not take into account the possible reverse complement compositional asymmetry, which is related to origins of replication and direction of transcription [55], [56]. This compositional asymmetry is a common feature of higher eukaryote genomes, especially in tests with relatively small (less than 1 Mb) windows. A slight modification of our calculations allows the consideration of this factor. Specifically, for chosen sequences *S*_1_ and *S*_2_, the calculated difference (say, *d*_12_) can be complemented by score *d*_12_, using the CS-distance between strand *S*_1_ and the complementary to *S*_2_ strand taken in the reverse direction. In the case of *S*_2_ strand symmetry, *d*_12_ and *d*_12_′ are close to each other; their difference will point to *S*_2_ asymmetry. The same calculations are conducted with a complementary to *S*_1_ strand taken in the reverse direction, resulting in *d*_1_′_2_ and *d*_1_′_2_′. Thus, *d* = min (*d*_12_, *d*_12_′, *d*_1_′_2_, *d*_1_′_2_′) is employed as CS distance.

### Sequence heterogeneity

We consider three types of sequence heterogeneity: compositional, organizational, and total, which is a combination of the first two types. The *compositional heterogeneity* score H_c_ is a measure of variation in nucleotide composition along the sequence. We calculate H_c_ as a median difference in GC content between equal-length segments (due to rather strong asymmetry of the distribution of this value). We can disregard “second-order” differences in the within-strand contents of G vs. C and A vs. T due to the rather strict correspondence of strand composition to the second Chargaff parity rule [57], [58]. *Organizational heterogeneity* (H_o_) reflects the differences in oligonucleotide structures along the sequence, which can be estimated by CS comparisons. Obviously, nucleotide composition of the compared sequences affects the oligonucleotide frequencies found in these sequences. The simplest way to assess the target sequence for H_o_ not mixed with H_c_ is to include in the CS analysis only pairs of segments with similar GC content although not all possible pairs of segments will then be included in the calculation. *Total heterogeneity* (H_t_) is calculated over all pairs of segments comprising the target sequence and is affected by both compositional and organizational differences between these segments.

To evaluate heterogeneity of a genome, chromosome or any other sequence, we subdivided it into equal lengths not overlapping 100 kb segments (although other sizes can also be used) and for each such segment calculated its GC content and CS. Median values of GC differences and CS distances calculated over all pairs of segments were used as assessments of H_c_ and H_t_, correspondingly. The median value of CS distances across pairs with zero or small GC differences was used as an estimate of H_o_. To evaluate compositional and organizational differences between two sequences (e.g., two chromosomes), referred to as inter-sequence heterogeneity, we calculated the GC differences and CS distances between each segment of one versus each segments of the other sequence.

**Figure 1:**
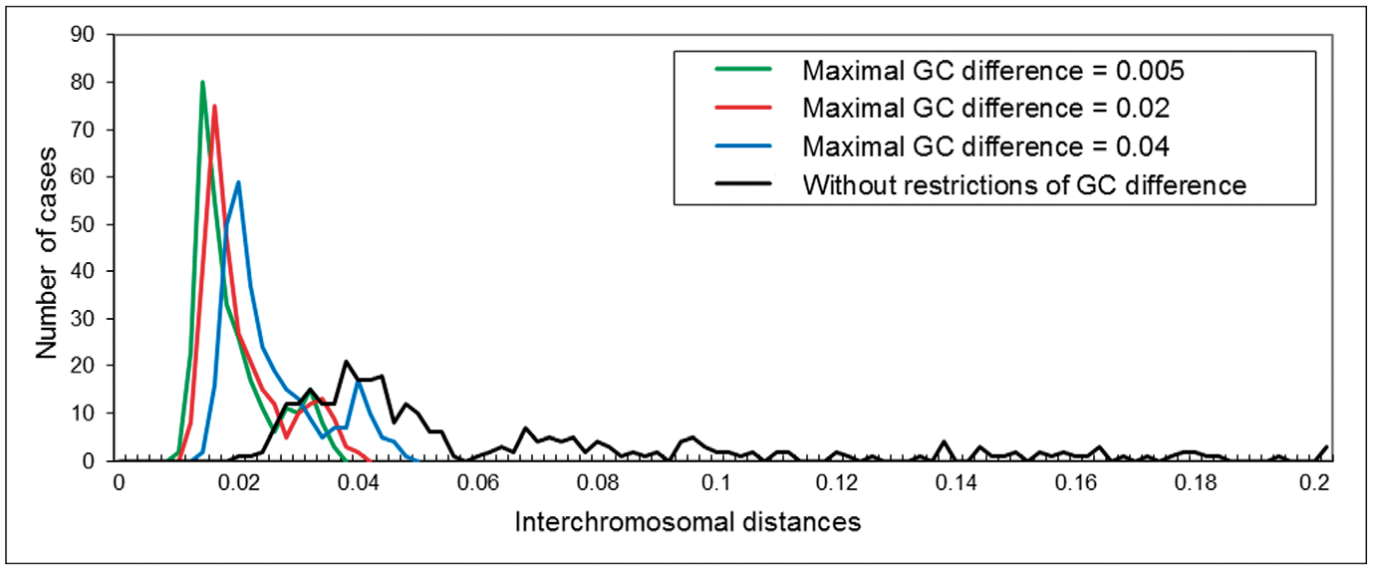
The distribution histogram of human median inter-chromosomal CS-distances obtained with different permitted maximal levels of GC differences between scored 100 kb segments. The inter-chromosomal CS-distances obtained without restriction on inter-segmental GC differences contain both compositional and organizational components of inter-chromosomal heterogeneity (thereby coinciding with H_t_). The stricter conditions on ΔGC reduce the influence of H_c_ on the estimate of H_o_. All ΔGC thresholds that are ≤0.02 show just slightly different distributions of human inter-chromosomal H_o_. Therefore, we believe that ΔGC≤0.02 condition permits us to assess organizational inter-chromosomal heterogeneity without the influence of compositional heterogeneity.

To determine the acceptable level of inter-segmental GC difference in H_o_ calculations, we compared the distribution of inter-chromosomal CS distances as a function of GC difference (ΔGC). It appeared that ΔGC≤0.02 provides sufficient stability of the distribution, i.e., it does not change considerably with further decreases of ΔGC (Fig. 1). Moreover, using a smaller ΔGC means a reduction of the number of analyzed pairs of segments. Thus, across the human chromosomes, about 26% of segment pairs participate in the analysis of organizational heterogeneity in using ΔGC≤0.02 and only 12% and 6% in cases of ΔGC≤0.01 and ΔGC≤0.005, respectively. On average, for the analyzed vertebrate genomes, using the condition ΔGC≤ 0.02 provides participation of 40% of segment pairs in H_o_ analyses (range: from 23% for pig *Sus scrofa* to 74% for tarsier *Tarsius syrichta*). Similar results were obtained with 50 kb and 500 kb segments (not shown). Therefore, in all subsequent tests, we employed the condition ΔGC≤0.02.

### Dependence of intra- and inter-sequence heterogeneity on the set of words

We tested a few different unbiased sets of words and found that intra-and inter-sequence heterogeneity scores of entire genomes, chromosomes, or chromosome segments do not depend on the set used.

## Results

### Intragenomic heterogeneity

**Figure 2:**
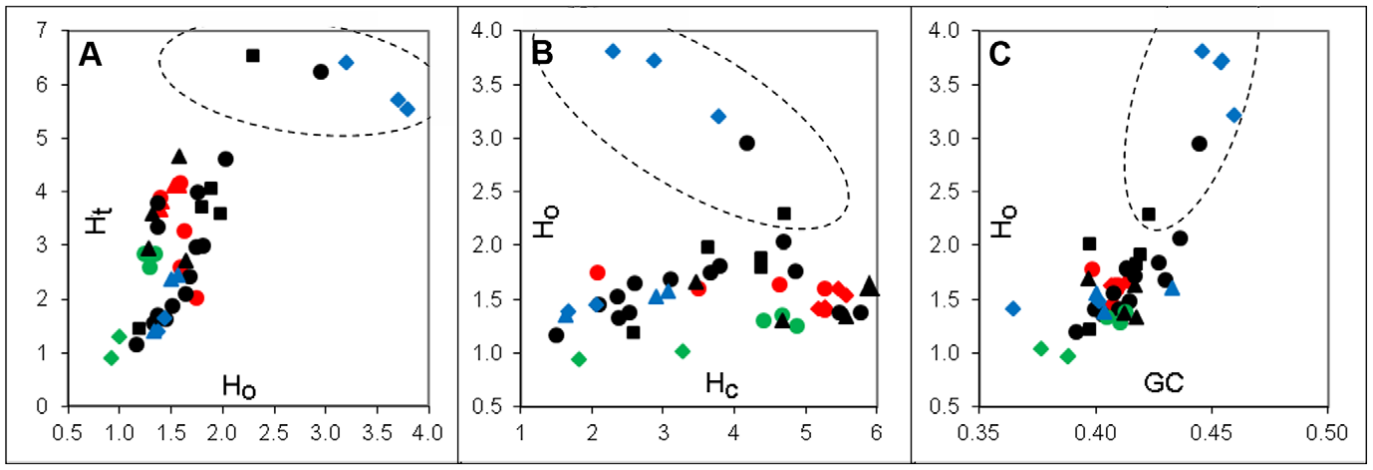
Intragenome heterogeneity and relationships between total and organizational (A), organizational and compositional (B) heterogeneity types, and organizational heterogeneity and genome GC content (C). Every series corresponds to one species group: ⧫ apes; ▴ other primates; ▪ Rodents; ▴ Ungulates; • other mammals; ⧫ Marsupials; • Birds; ⧫ Fishes; ▴ other vertebrates. The species surrounded by a dashed ellipse appeared to be outliers due to their organizational heterogeneity (see explanation in the text).

**Figure 3:**
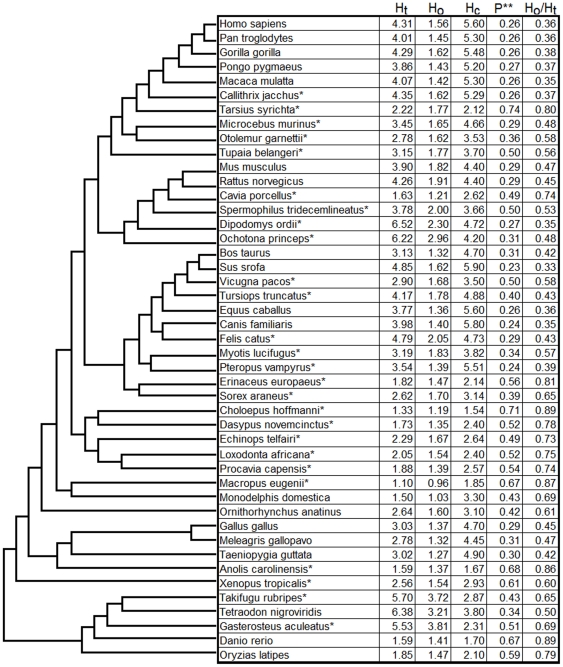
Whole-genome compositional, organizational and total heterogeneity for 45 vertebrate species (•10^−2^). The deviation of the sampled median values is less than 0.0001 in all genomes for each of the three scores. Notes: * – incomplete genome sequences. We evaluate H_o_ by the same method as H_t_, so one can directly compare H_o_ and H_t_ in corresponding columns of the table unlike H_c_, which is calculated as a median difference in GC content between equal-length segments, so that corresponding H_c_ values are not directly comparable with H_o_ and H_t_. P ** – The part of included segment pairs (i.e., those with very similar GC content) out of the total number of possible segment pairs.

First, we analyzed different types of whole-genome heterogeneity: compositional (H_c_), organizational (H_o_), and total (H_t_) for 45 fully sequenced vertebrate genomes from different taxonomic groups. For every genome sequence, subdivided to 100 kb segments, we calculated CS distances and GC differences for all segment pairs and estimated H_c_, H_o_, and H_t_ (see section Methods). All three heterogeneity types varied more than 4–5-fold across species (Figure 2). Maximal H_t_ values were observed within fishes, apes, and rodents; fishes and rodents showed maximal H_o_ as well, while in apes H_o_ was relatively small (36–38% of their H_t_). Minimal H_t_ and H_o_ values were found in marsupials, but H_o_ of marsupial genomes reached almost 80% of H_t_, which is the maximal value among all analyzed genomes. The highest variability of all heterogeneity values was found in fishes, rodents, and “other mammals” group; ape and bird genomes turned out to be very similar to each other. Five species selected for their highest H_t_ also appeared to have the highest H_o_ but not H_c_ (Fig. 3); it is interesting that three of these species were fishes (*Gasterosteus aculeatus, Takifugu rubripes,* and *Tetraodon nigroviridis*), two others were mammals: pika (*Ochotona princeps*) and kangaroo rat (*Dipodomys ordii*). A relatively high correlation between H_t_ and H_c_ scores was observed in vertebrate genomes (Spearman rank correlation coefficient ρ = 0.67) and between H_t_ and H_o_ (ρ = 0.69), while no correlation was found between H_o_ and H_c_ (ρ = 0.08). According to linear multiple regression analysis, a combination of both variables (H_c_ and H_o_) explained R^2^ = 0.94 of variation in H_t_ between species, while the separate effects of H_o_ and H_c_ on H_t_ were R^2^ = 0.53 and 0.35, respectively. Despite considerable dependence of H_o_ and H_t_ on genome average GC content (ρ = 0.63 and 0.61, respectively), in the regression analysis the variable “GC content” proved unimportant.

The previously mentioned characteristics of intragenomic heterogeneity automatically included variation within and between chromosomes. However, the segments with different compositional and organizational characteristics can be more or less proportionally represented in different chromosomes of the same genome. By “CS-genomic states” one, therefore, may consider groups of genomic segments with similar nucleotide composition and similar organization, i.e., with small H_t_, H_o_, and H_c_ within the group. Presumably, each of the five isochore groups in the human genome, in principle, may be further split into sub-groups with small H_o_ within and high H_o_ between the sub-groups. It would be natural to refer to these sub-groups as *CS-genomic states* or *organizational isochores*. If these hypothetical CS-genomic states are distributed proportionally between chromosomes, intra-chromosomal and inter-chromosomal heterogeneity (i.e., compositional and organizational differences over all pairs of chromosomes) will be close to the whole-genome heterogeneity. Alternatively, if some genomic state is heavily overrepresented in one or more chromosomes, intra-chromosomal heterogeneity of the corresponding chromosome (chromosomes) should decrease while its average inter-chromosomal distances to the remaining chromosomes should increase.

**Figure 4:**
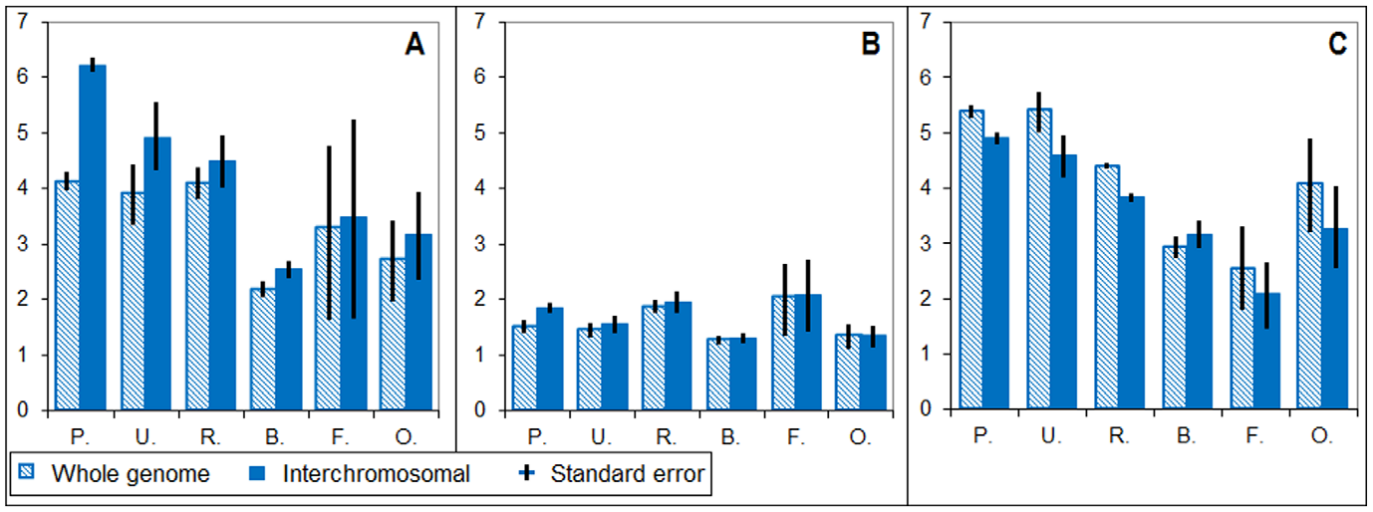
Whole-genome and inter-chromosomal heterogeneity scores H_t_, H_o_, and H_c_ for different groups of analyzed vertebrate genomes: (A) total heterogeneity, (B) organizational heterogeneity, (C) compositional heterogeneity; Legends: P. primates, U. ungulates, R. rodents, B. birds, F. fishes, O. other vertebrates; black bars correspond to standard error.

We have calculated the median inter-chromosomal H_t_, H_o_ and H_c_ (i.e., total, compositional, and organizational differences for all pairs of chromosomes) and compared them with corresponding whole-genome heterogeneities for the vertebrate species, which were assembled into chromosomes at the moment of analysis (19 species). It was found that average inter-chromosomal H_t_ and H_o_ values are greater than the corresponding whole-genome H_t_ and H_o_ values for all analyzed genomes, presumably reflecting non-proportional distribution of CS-genomic states between chromosomes (Fig. 4a). On the contrary, average inter-chromosomal H_c_ was less than whole-genome H_c_ for all genomes except for birds (Fig. 4b). The greatest differences between average inter-chromosomal and whole-genome H_t_ and H_o_ were observed for primate genomes, presumably reflecting a greater chromosome structural specialization reached during the evolution of this group.

### Heterogeneity of the coding and non-coding parts of genomic DNA

The genome is a mosaic of sequences with different compositional, organizational, and functional features. The organizational isochores are not available at this stage of our study, but we can investigate the possible relationship of heterogeneity of genomic CS states with its diverse functional features. The simplest separation of genome sequence into functional fractions is by considering coding DNA and repetitive sequences. By “coding” sequences we mean the genes (both exonic and intronic parts), pseudogenes, RNA-coding DNA, etc. From the noncoding part of the genome, we extracted all known repetitive elements (using RepeatMasker as a tool); the remaining part of the noncoding DNA was referred to as *genome dark matter* (GDM). The analysis of every genomic fraction was conducted after isolating of corresponding sequences. Thus, to analyze the heterogeneity of GDM we first masked the repetitive elements and coding DNA in the sequence. The remaining parts of sequence were concatenated and subdivided into 100 kb segments (windows).

Three heterogeneity types: compositional, organizational, and total, of six vertebrate genomes (three mammalian, one avian, and two fish species) were assessed separately in three genome subspaces (genic, repetitive elements, and GDM) and in the whole sequence. Compositional heterogeneity of each of the three genomic fractions in the analyzed species was lower than the whole-genome compositional heterogeneity, with the only exception of the zebrafish genome, where H_c_ in the repetitive DNA was higher than in the whole genome (Table 2). However, among the compared six species the GC-poor zebrafish genome showed the lowest values of H_c_ in all of its functional fractions.

**Table 2:** Heterogeneity of different genome fractions (·10^−2^).

	H_t_ *	H_o_ *	H_c_ *	H_o_/H_t_	H_t_ *	H_o_ *	H_c_ *	H_o_/H_t_	H_t_ *	H_o_ *	H_c_ *	H_o_/H_t_
	*Homo sapiens*				*Mus musculus*				*Canis familiaris*			
Full sequence	4.31	1.56	5.60	0.36	3.90	1.82	4.40	0.47	3.98	1.40	5.80	0.35
Coding DNA	5.05	1.76	4.55	0.35	4.78	2.23	3.96	0.47	5.22	1.59	5.10	0.30
Repeats	4.34	1.94	3.19	0.45	3.39	2.11	2.22	0.62	2.72	1.62	2.69	0.60
GDM	3.23	0.83	5.22	0.26	3.13	1.53	3.32	0.49	4.61	1.07	5.59	0.23

	*Gallus gallus* **				*Danio rerio*				*Tetraodon nigroviridis*			
Full sequence	3.03	1.37	4.70	0.45	1.59	1.41	1.70	0.89	6.38	3.21	3.80	0.50
Coding DNA	2.19	1.24	2.90	0.57	1.41	1.29	1.19	0.91	7.09	3.40	3.24	0.48
Repeats	3.47	2.35	2.33	0.68	2.27	1.98	1.79	0.87	4.58	2.88	1.77	0.63
GDM	5.21	1.71	3.62	0.33	0.95	0.89	0.98	0.94	5.20	2.72	2.73	0.52

The deviation of the sampled median values is less than 0.0001 in all genomes for each of the three scores.

*H_c_, H_o_ and H_t_ – compositional, organizational and total heterogeneity scores. Note that H_c_ is calculated as median of differences in GC content while H_t_ and H_o_ scores are evaluated based on comparison of compositional spectra (see section Methods);

**only large chromosomes 1–8 were taken into account.

Unlike compositional heterogeneity, the organizational heterogeneity of repetitive and coding DNA in three mammalian genomes was higher than organizational heterogeneity of whole-genome sequences, while their GDM showed minimal H_o_ among all genome fractions. In the chicken genome, minimal value of H_o_ was found in coding DNA, whereas its repeats and GDM were more heterogeneous than the whole-genome sequence. Two analyzed fish genomes did not show similar differentiation of H_o_ among their genomic fractions: in zebrafish, the most heterogeneous genome part was repetitive DNA whereas in pufferfish – it was coding DNA. Interesting that the ratio of H_o_/H_t_ was maximal in the repetitive DNA in all genomes, excluding zebrafish, where it was minimal in repeats and maximal in GDM. However, high H_o_/H_t_ is characteristic of the zebrafish genome and all three of its fractions, caused by combined effect of relatively high H_o_ and relatively low H_c_ of corresponding sequences.

**Figure 5:**
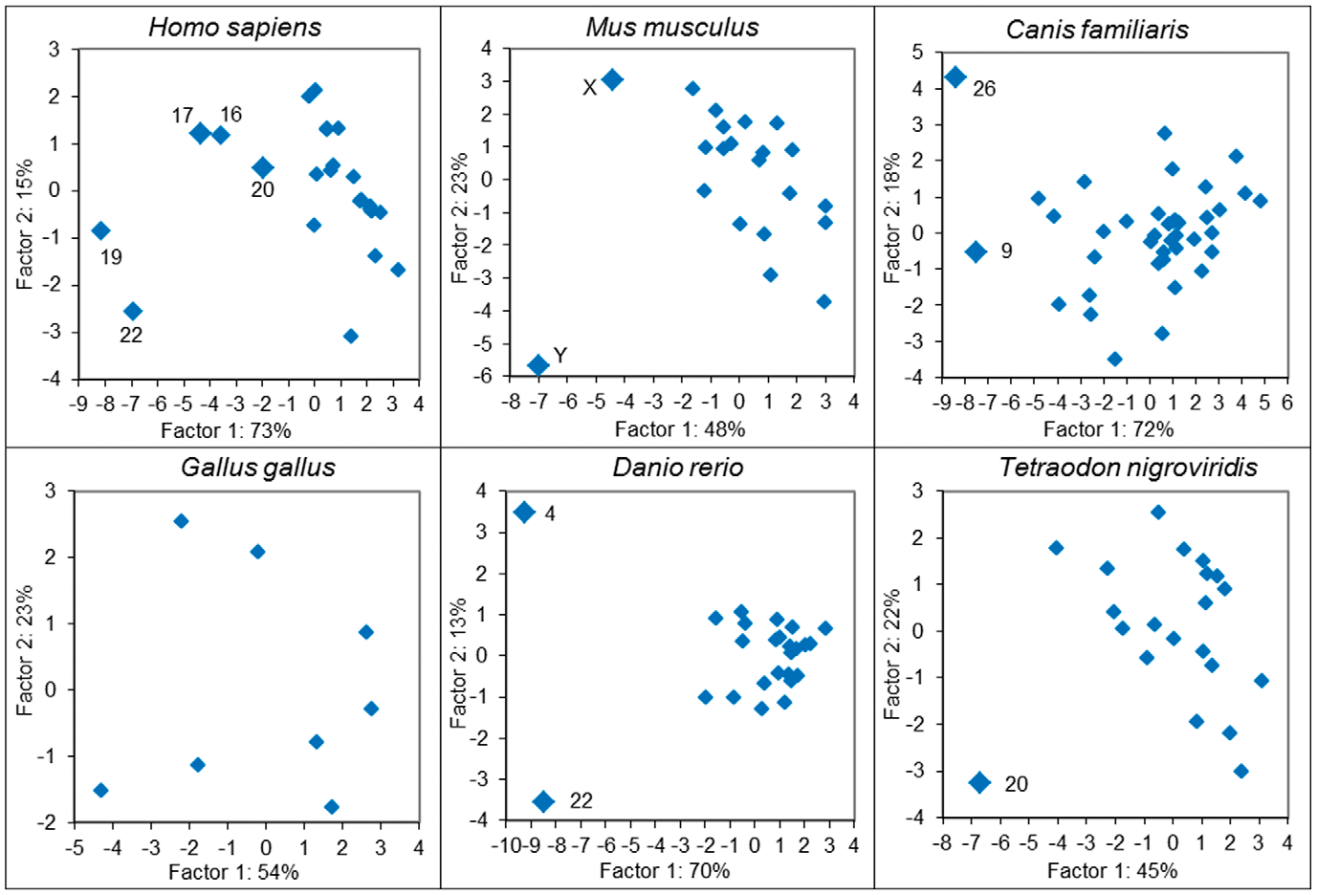
Principal component analysis of vertebrate chromosomes for H_o_ and H_c_ scores evaluated in three genome fraction (coding sequences, repetitive elements, and GDM).

The previously mentioned results on the variation of H_t_, H_o_, and H_c_ in whole-genome and inter-chromosomal comparisons (see Fig. 4) point to a possible non-proportional distribution of CS-genomic states between chromosomes. Thus, we may expect to find outlier chromosomes with respect to heterogeneity parameters. Analogously to the whole-genome analysis, we estimated inter-chromosomal H_o_ and H_c_ components for the three genomic factions of the six vertebrate species and complemented these scores by corresponding intra-chromosomal H_o_ and H_c_ components. Based on the principal component analysis (PCA), an assessment of between-chromosome variation for each of the six species was conducted using the first two PCA components, which explained 67–90% of the variation in the 12-dimension space (H_o_ and H_c_ scores of intra- and inter-chromosomal heterogeneities for the three genomic factions). One or two outlier chromosomes were found in each of the analyzed mammalian and fish genomes (Fig. 5). Note, that dog outlier chromosomes include large regions syntenic with human outlier chromosomes: gene order of dog chromosome 9 is close to that of human chromosome 17; third of dog chromosome 26 is syntenic with human chromosome 22; and all these chromosomes are relatively GC-rich. In mouse, the outliers appeared to be sex chromosomes. We did not found in the literature any specific features of fish chromosomes that appeared as outliers in our analysis.

**Figure 6:**
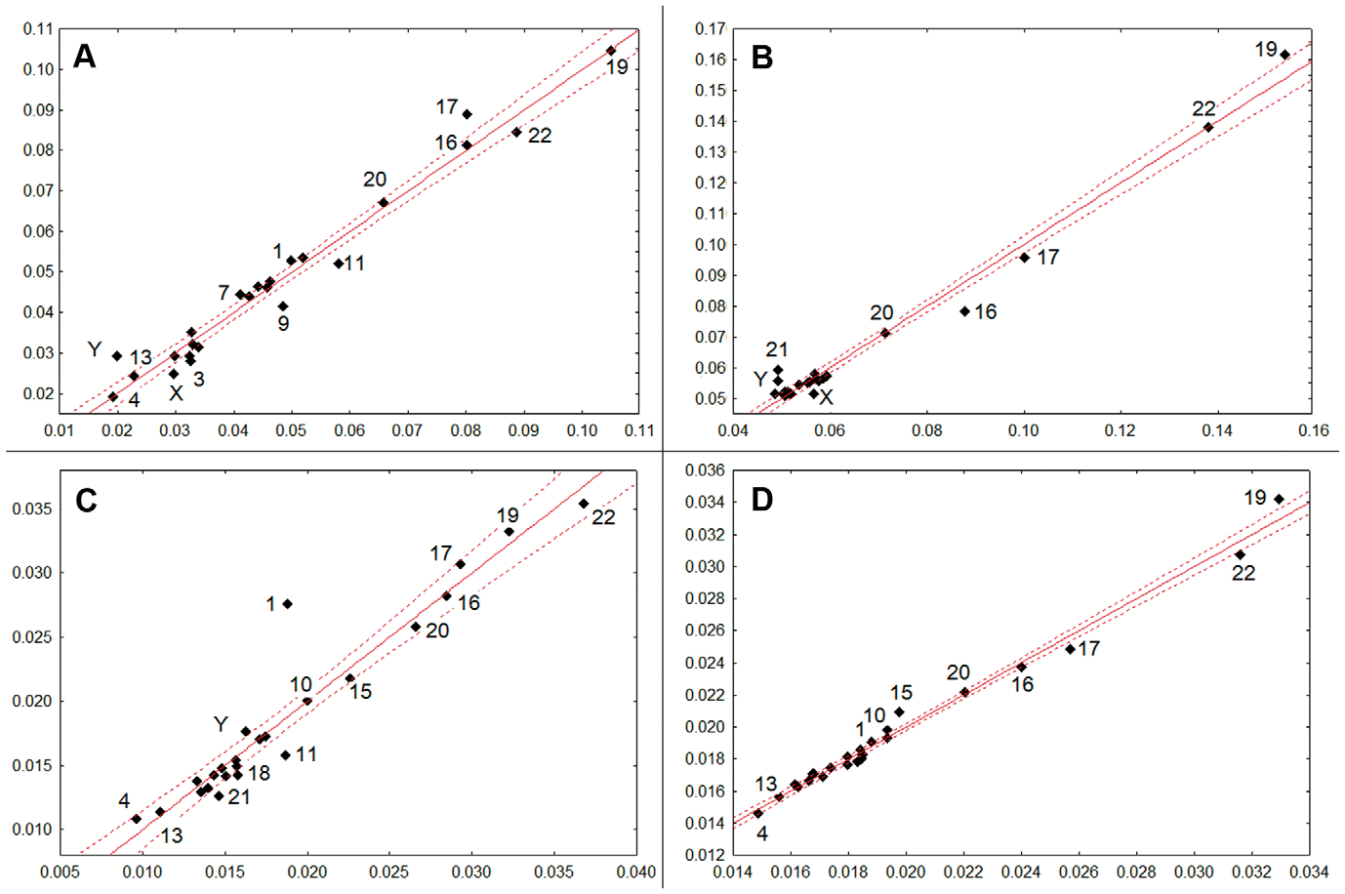
Predicted and observed values of human intra- and inter-chromosomal heterogeneity estimates. Axes X: predicted values, axes Y: observed values. (A) intra-chromosomal H_t_; (B) average inter-chromosomal H_t_; (C) intra-chromosomal H_o_; (D) average inter-chromosomal H_o_.

Additional details on relationships between heterogeneity components for different genome fractions can be seen in the example of human chromosomes. We were interested in assessing how the total and organizational intra- and inter-chromosomal heterogeneity depend on H_o_ and H_c_ scores of the chromosomal sub-sequences (coding, repetitive and GDM) and their GC content. For each human chromosome, intra- and inter-chromosomal H_o_ and H_c_ scores for each of the three sub-sequences were calculated together with their GC content, as potential “predictors” for whole-chromosome total and organizational heterogeneity. Using stepwise linear regression analysis, the significant predictors were determined separately for intra- and inter-chromosomal H_t_ and H_o_ characteristics (Fig. 6 and Table 3). Combinations of only 2–3 variables out of 9 explained 0.98 of the variation in intra- and inter-chromosomal H_t_, and 0.9 and 0.99 of variation in their intra- and inter-chromosomal H_o_, correspondingly. An interesting fact is that GC content of GDM proved to be important in explaining both intra- and interchromosomal H_o_ variation, while in the analysis of corresponding H_t_ values, CG contents of all three sub-sequences were not significant. H_c_ of the repetitive DNA appeared a significant predictor for both characteristics of inter-chromosomal heterogeneity, total and organizational. However, for the organizational heterogeneity, the other score of repetitive DNA, H_o_, was even more informative. In turn, H_o_ of coding DNA was significant for both total and organizational intra-chromosomal heterogeneity. H_c_ of coding DNA was important only for total intra-chromosomal heterogeneity, but its importance there was much higher than the importance of H_o_ of coding DNA. The H_o_ score of GDM was important for intra- and inter-chromosomal whole-sequence total heterogeneity.

**Table 3:** The most significant predictors of intra- and inter-chromosomal heterogeneity scores H_t_ and H_o_.

Intrachromosomal H_t_	t(20)	p-value	Interchromosomal H_t_	t(21)	p-value
Coding DNA H_c_	11.34	<5×10^−7^	Repetitive DNA H_c_	12.35	<5×10^−7^
GDM H_o_	6.78	<5×10^−6^	GDM H_o_	5.19	<4×10^−5^
Coding DNA H_o_	3.78	0.001			
*Adjusted R^2^*	0.98		*Adjusted R^2^*	0.98	

Intrachromosomal H_o_	t(21)	p-value	Interchromosomal H_o_	t(20)	p-value
GC content of GDM	3.77	0.001	Repetitive DNA H_o_	8.95	<5×10^−7^
Coding DNA H_o_	3.05	0.006	Repetitive DNA H_c_	4.65	<2×10^−4^
			GC content of GDM	2.15	0.043
*Adjusted R^2^*	0.91		*Adjusted R^2^*	0.99	

### Effect of GC content on sequence organization

**Figure 7:**
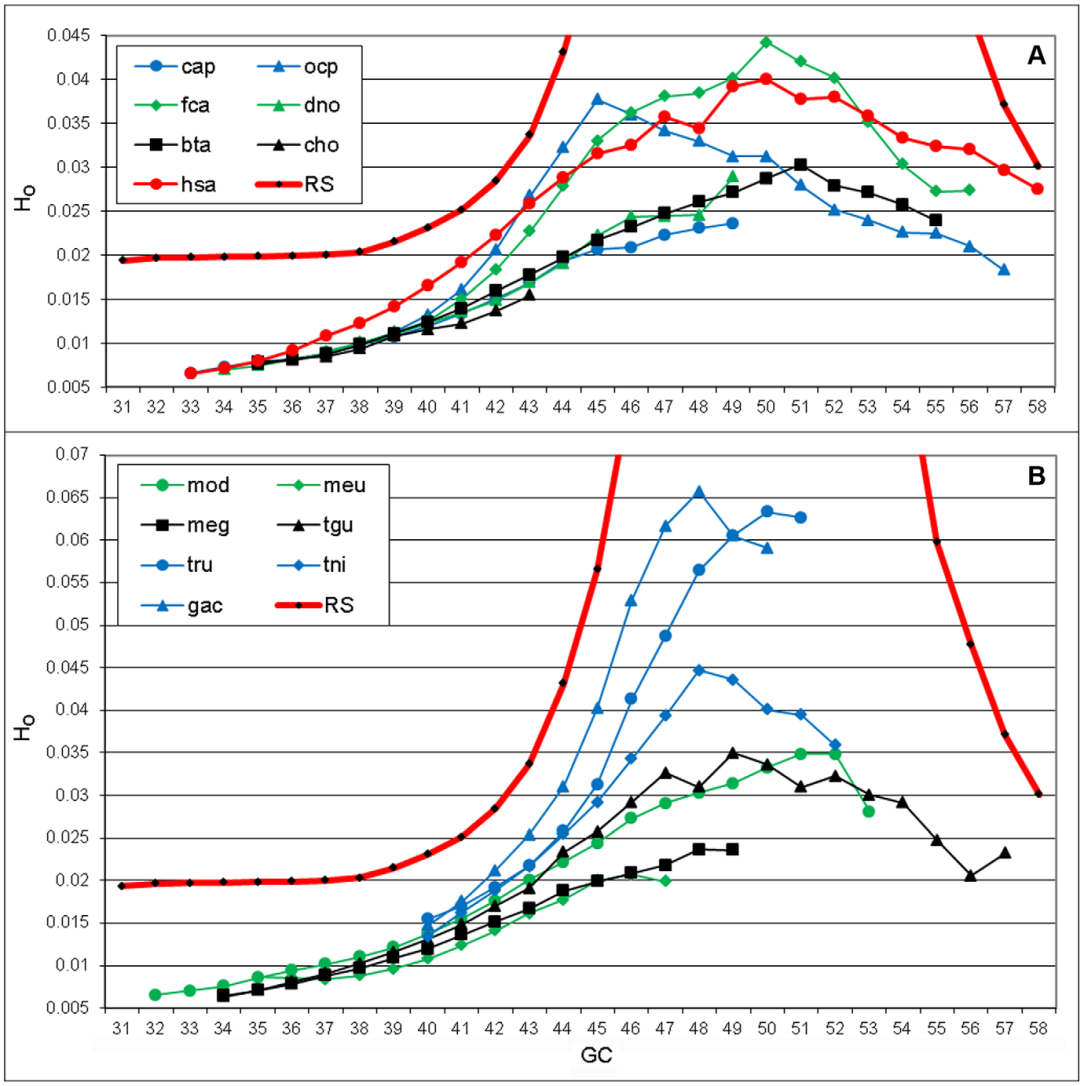
Organizational heterogeneity of natural sequences of different vertebrate genomes. (A) mammals, (B) non-mammalian species. Legend: **cap** guinea pig (*Cavia porcellus*), **dno** armadillo (*Dasypus novemcinctus*), **gac** stickleback (*Gasterosteus aculeatus*), **meu** wallaby (*Macropus eugenii*), **mod** opossum (*Monodelphis domestica*), **ocp** American pika (*Ochotona princeps*), **tru** fugu (*Takifugu rubripes*), **tni** pufferfish (*Tetraodon nigroviridis*), **hsa** human (*Homo sapiens*), **bta** cow (*Bos taurus*), **cho** sloth (*Choloepus hoffmanni*), **fca** cat (*Felis catus*), **meg** turkey (*Meleagris gallopavo*), **tgu** zebra finch (*Taeniopygia guttata*), **RS** reshuffled sequence.

The previously mentioned comparisons of vertebrate genomes revealed positive correlation between GC content and H_o_ values (see Fig. 2c). Is this tendency of genome organization also expressed on the intra-genome level (between segments of the same genome)? To address this question, we analyzed 14 vertebrate genomes from diverse taxonomic groups. As before, we subdivided the whole-genome sequence of each species into segments of 100 kb length and classified the segments, regardless of their chromosomal residence, into groups according to GC content (with 1% of GC content width per group). The small groups from the tails of GC distribution were removed from the analysis. In 3 out of 14 tested species the highest H_o_ was observed in segments with GC≈0.5 and 6 other species showed the highest H_o_ at GC = 0.48–0.50 or 0.50–0.52 (Fig. 7). The pika genome had an “extreme” point at CG = 0.45 and the wallaby, at CG = 0.46. Three other species did not have any “extreme” point and showed the highest H_o_ in their GC-richest segments. Organizational heterogeneity of GC-rich segments in fish genomes was higher than in corresponding groups of mammalian, birds, and marsupial genomes.

In previous studies, we employed a reshuffling test to assess the significance of genome organization compared to random sequences with the same nucleotide abundances [59]. This test was applied here (with some modifications) to vertebrate species in order to assess the nonrandomness of genome organization in terms of compositional spectra and its dependence on GC content. To conduct reshuffling, each letter of segment sequence, starting from the first position, was swapped with a letter from a randomly selected position within the segment. This procedure returns a new sequence of the segment with unchanged letter abundances but a random letter order. Therefore, in addition to the initial (natural) sequences, we calculated the heterogeneity within each group of segments using the reshuffled sequences.

As expected, after reshuffling, the groups of segments with the same GC content showed very similar values of H_o_, no matter to which genome they belonged. Likewise, they showed a strong correlation between their H_o_ and deviation of GC content from 0.5. The following straightforward argument explains this fact. For simplicity, we assume that %A = %T and %C = %G in single-strand DNA, in accordance to the Chargaff's second parity rule [56], [57]. Violation of this rule was found in some regions related to replication origin, gene location and transcriptional hot spots [60], and the calculations below can be extended to such cases with qualitatively similar results.

Let the frequencies of letters A and T in the sequence be equal to *n*_1_/2 = *p*_1_, and letters C and G to *n*_2_/2 = *p*_2_ (we assume that *n*_1_ and *n*_2_ are even); the length of the entire sequence is *n*_1_+*n*_2_ = *n.* Thus, *p*_1_+*p*_2_ = *n/*2. It is easy to calculate the number *N* of all possible different sequences with these parameters:
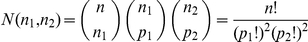
For symmetry, we write the value *N* as a function of two variables, although it is clear that *n*_1_, for example, can be directly calculated from the values *n* and *n*_2_. It is easy to show that this expression reaches its greatest value at *n*_1_ = *n*_2_. Using the known asymptotic approximations for the binomial coefficients we can calculate
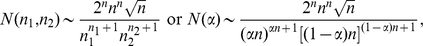
where *α* is GC content of segments.

As follows from these calculations, sequences with GC content close to 0.5 have a much higher potential of variation compared to sequences with other GC contents. In accordance with the used method of CS-distance calculation, heterogeneity (i.e., median CS distance) of large enough group of random segments with strictly identical proportions of all four nucleotides (25% of each) should be equal to 0.5 (i.e., rank correlation coefficient between compositional spectra of such segments should be equal or close to zero). All random segments with deviations from identical proportions of all four nucleotides, such as different CG content of analyzed segment groups, should result in spectra with *d*<0.5 (i.e., with positive rank correlation) because of overrepresentation of certain nucleotide(s). In fact, H_o_ of the reshuffled sequences for the analyzed groups with GC≈0.5 ranged in different genomes from 0.33 to 0.42, presumably due to small-scale variability of GC in these groups (from 0.495 to 0.505) and possible violations of the Chargaff's second parity rule for either G vs. C, or A vs. T, or both.

In general, the natural sequences of the tested species proved to be much less heterogeneous than corresponding random sequences with the same GC content, reflecting the high level of genome organization. Moreover, the difference between the reshuffled and natural sequences was found to be maximal for the groups of sequences with GC in the range 0.49–0.51 and decreased with the increase in |GC-0.5|. This may indicate that anti-entropic evolutionary forces shaping the genome organization have imposed stronger impact on regions with equal abundances of all four nucleotides compared to other regions.

**Figure 8:**
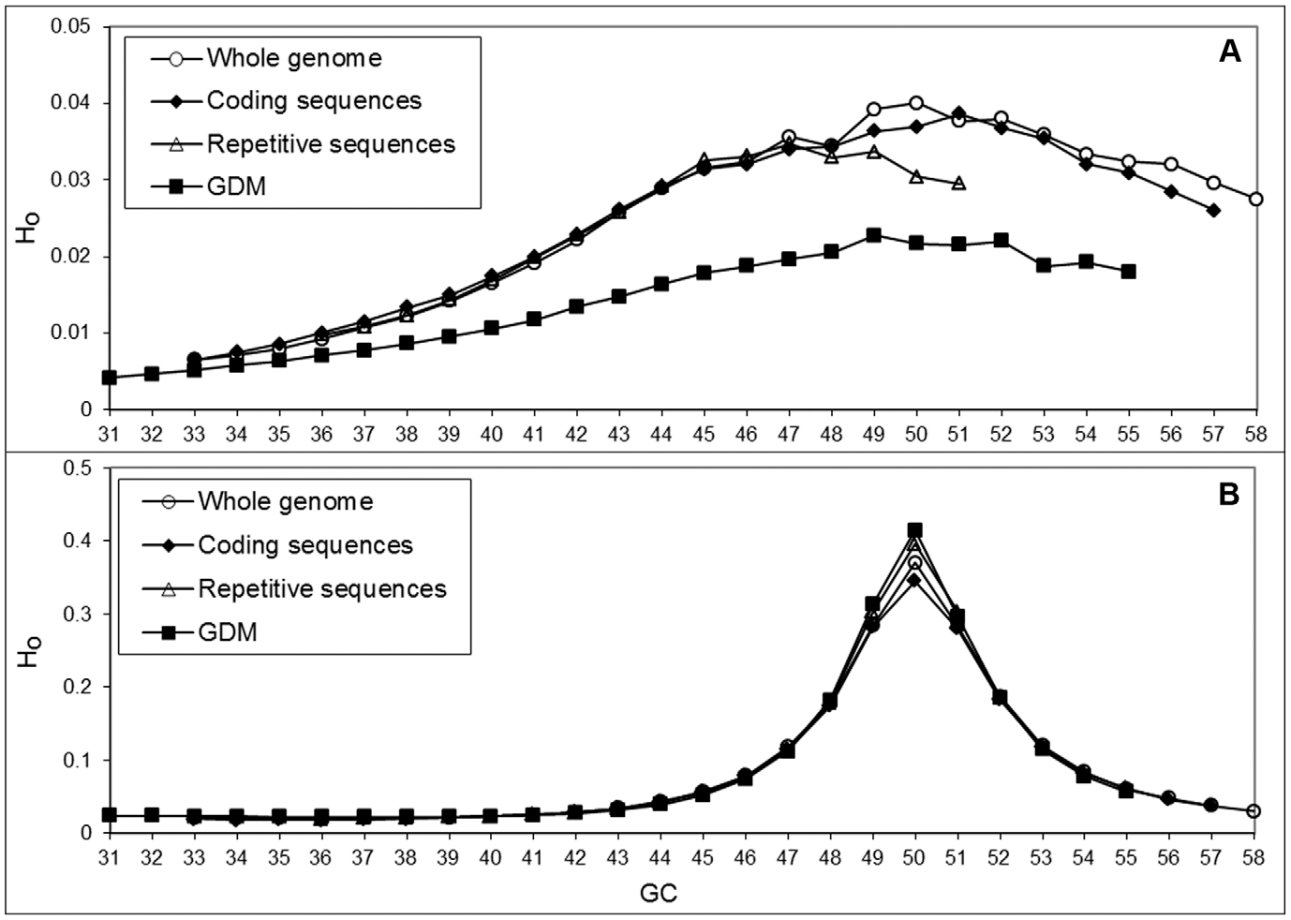
Organizational heterogeneity of natural sequences of different genome fractions and corresponding reshuffled sequences with varied GC content: (A) natural sequences, (B) reshuffled sequences.

In addition to above analysis of deviation of the full sequence of human genome from corresponding random sequences for each GC window, the same analysis was conducted for separate genome fractions: coding, repetitive sequences, and GDM. Due to different compositional limitations we obtained different numbers of GC-groups: 23 for the coding DNA (CG range from 0.33 to 0.55), 15 for repetitive DNA (CG range from 0.36 to 0.50), and 20 for the GDM (CG range from 0.31 to 0.50). As before, natural sequences were considerably less heterogeneous than the reshuffled ones; the largest difference was observed in GDM, implying that GDM is *the most organized DNA fraction in the human genome* (Fig. 8a). The observed differences between H_o_ values of reshuffled sequences of separated genome fractions (Fig. 8b) can presumably be explained by varied deviations from Chargaff's parity rule in these fractions.

## Discussion

The problem of genome heterogeneity has been widely discussed in the last decade [1], [8], [20], [61]–[65]. A considerable part of the studies is devoted to GC heterogeneity and its relationship with the functional and evolutionary heterogeneity. GC content displays a wide interspecific variation as well as high within-genome heterogeneity, especially in mammals, where it is presented in the form of GC isochores [66]. GC content is strongly correlated with biological features of the genome organization such as distribution of various classes of repeated elements, gene density, level and tissue-specificity of transcription, and mutation rate. The available data point to a strong correlation between GC content and recombination. This connection may be caused by increased recombination in GC-rich regions. An alternative assumption is that meiotic recombination machinery is responsible for the evolution of GC distribution due to the bias of mismatch repair within the gene conversion tracts in favor of GC, which preferentially converts A/T into G/C at sites that are heterozygous for AT and GC - the biased gene conversion hypothesis [67], [68]. A possibility that another factor, affecting both the evolution of intragenomic heterogeneity in GC content and recombination, is responsible for their positive association cannot be excluded.

We used the average difference of GC content between genome segments as an indicator of its compositional heterogeneity, which can be approximately compared with GC-profiles discussed in previous publications. Our results on interspecific variation of compositional heterogeneity, as expressed by the differences in H_c_, in general correspond well to previous studies based on assessment of isochore structure of vertebrate genomes. In particular, closely related primate genomes displayed similar and relatively high values of compositional heterogeneity H_c_ corroborating with similar isochore patterns [69]. Generally, genomes of cold-blooded vertebrates are less heterogeneous than genomes of warm-blooded vertebrates in terms of H_c_ (our results) and isochore organization [69]. Although bird genomes have wider GC profiles due to the presence of very GC-rich H4 isochores [70], in terms of H_c_ they are less heterogeneous than primate genomes. The last discrepancy can be explained by a small proportion of GC-rich segments in bird genomes and their relatively weak contribution to the whole-genome H_c_ estimate.

Organizational heterogeneity of the studied genomes also varied among analyzed species: relatively high values were observed in 3 out of 5 fish genomes and in 2 out of 32 studied mammal genomes, while the lowest values were found in marsupials, sloth, and guinea pig (see Table 1). It is noteworthy that similar values of H_o_, H_c_, and H_t_ are displayed by closely related species in two groups of organisms (birds and apes), while in two other groups (rodents and fishes) all heterogeneity types were more variable. In lower (non-human) primates, ungulates, and marsupials, low intra-group variation of H_o_ values was observed together with considerable variation of H_c_ and H_t_, presumably reflecting higher functional importance of organizational patterns in genome structure and evolution. Compositional and organizational similarity of different segments of the same genome indicates the existence of genome structural patterns.

Similar to H_c_ values, the absolute H_o_ values are relatively small, especially when compared to corresponding reshuffled sequences. However, their variability among genomes, chromosomes or chromosome segments may be an important source of information for the investigations of genome structure, functions and evolution. We found that intra-genome variability of the compositional and organizational patterns may contain sufficient information for revealing conserved parallelism upon a modified dot-plot genome comparison based on compositional spectra analysis [48]. Remarkably, GDM by itself allows to get a rather good coverage in such dot-plot comparisons between humans and apes [Kirzhner et al. unpublished results], implying evolutionary conservation in intragenomic variation of compositional and organizational patterns that extends beyond the genic space. Evolutionary conservation of noncoding DNA among vertebrates was intensively studied during the last decade [52], [71]–[73]. It was found that some noncoding elements are even more conserved than genes. Although the location of a part of these elements in the intergenic regions does not necessarily point to their role as gene regulatory elements, many examples are known in which this is indeed the case. Some estimates indicate that the vertebrate-specific ultraconserved noncoding regions may be under selection comparable to or stronger than selection on protein-coding regions [74]. The total length of evolutionary conserved noncoding DNA is about 2.5–5% of the mammalian genome length, whereas our definition of GDM leads to several-fold larger estimates, implying that evolutionary less conserved sequences may be the majority in GDM.

The results of multiple regression analysis of the whole-genome heterogeneity permits the conclusion that inter-specific variation of total genome heterogeneity characteristics as measured by CS analysis depends both on genome composition and organization. A similar effect was observed in the analysis of inter- and intra-chromosomal heterogeneities of different genomes, including the analysis of whole-chromosome sequences and their separate sub-sequences (coding, repetitive, and GDM sequences). This can be explained by assuming the existence of “organizational isochores”, i.e., segments with different organization within the compositional (GC) isochores. Similar ideas of organizational heterogeneity on a variable range of scales lower than those of GC ishochores were proposed earlier based on the concept of long-range correlations in DNA sequences [75]. The segments with different composition obviously have different spectra, but their organizations could not be fully compared by the proposed method. A CS comparison of sequences with the same (or similar) nucleotide composition allows detecting groups of segments with similar CS organization, which may be related to different structural, functional, and evolutionary features of genomic sequences.

The comparison of intragenome heterogeneity values with and without taking into account intrachromosomal heterogeneities suggests disproportional distributions of CS-genomic states between chromosomes. Moreover, PCA and multiple regression analysis reveals chromosomes dissimilarities of the same genome by their H_o_ and H_c_ values calculated on diverse genome fractions (see Fig. 5). Notably, for human genome, dissimilarities of the same chromosomes (16, 17, 19, 20 and 22) were observed by several authors. It was found that these chromosomes are GC-, CpG-, and gene-rich [76]–[78] and differ from other human chromosomes by enrichment with various repeats [77]–[80]. According to numerous publications, these chromosomes also differ by their structural and evolutionary features. Malcolm et al. [7] clustered human chromosomes into families according to genic mutation rates and noted a significant distinction of chromosomes 19 and 21 from other chromosomes. Surprisingly, our results on human inter-chromosomal organizational CS-heterogeneity (see Fig. 6) corroborate with inter-chromosomal heterogeneity with respect to the proportion of sequences under selection [81]. Authors marked chromosomes 19 and 22 as outliers due to relatively low proportions of both total and nonprotein-coding sequences under selection in spite of very high proportions of coding DNA in these chromosomes. Buschiazzo et al. [63] explain significant differences between human chromosomes related to the fraction of DNA alignable with other vertebrate genome sequences, which is lowest in chromosomes X, 19 and 22 and highest in chromosomes 13 and 18; this also implies dissimilarities in the rates of chromosome evolution. Correlation between chromosomal and regional variation in recombination rates and corresponding compositional features (GC isochores) of human chromosomes [17] fits well the biased gene conversion hypothesis [67].

Most aspects of intra- and inter-chromosomal heterogeneity correlate with chromosome GC content. For clarification of the influence of GC content on the sequence organizational heterogeneity, we compared heterogeneity of genome segments with different GC contents. It was found that organizational heterogeneity of segments increases with their GC content up to some “extreme point” and then decreases. This could be related to the expected relationship between the observed and potential variability of sequences as a function of GC content: higher variation is expected when GC is close to 0.5. However, in several cases, the greatest H_o_ values were found in segment groups with GC<0.5. An alternative explanation of greater organizational variability of CG-rich segments may be based on the fact that classical CG-isochores are not really compositionally homogeneous (i.e., GC content within isochores is variable). Moreover, GC-rich isochores are more compositionally heterogeneous than those that are GC-poor [10]. However, our “GC-slices” are much thinner than classical GC-isochores, and the variability of GC-content within every group of segments does not exceed 0.01; thus, different compositional heterogeneity of segments cannot be considered a cause of the observed differences in their organizational heterogeneity. Therefore, isochores of each GC range can be further classified into sub-sets, with similar CS patterns within the sub-sets and those that are dissimilar between sub-sets. Such sub-sets superimposed on the classical GC isochores can be referred to as “organizational isochores”.

High gene and SINE density is characteristic of GC-rich regions in most vertebrate genomes [81]–[82]. One may assume that a high level of organization of GC-rich regions (expressed as strong deviation from reshuffled sequences) is predetermined by the presence of well-organized coding DNA or highly repetitive sequences. However, the analysis of diverse fractions of the human genome (coding, repetitive sequences, and GDM) shows the same heterogeneity distribution pattern in each of these fractions (see Fig. 8a). Moreover, coding DNA appears to be the most heterogeneous fraction, whereas GDM, which does not have known genes and repeats (otherwise they would be masked by our masking pretreatment), shows the highest degree of organizational homogeneity, hence the highest deviation from the reshuffled (random) sequence. This may be partially explained by the presence of a fraction of highly conserved non-protein-coding DNA that in our analysis should be a part of GDM. We, therefore, speculate that the high conservation of numerous short intergenic sequences may be just the tip of the iceberg, namely, a more massive, albeit more fuzzy, organizational conservation (see also [73]).

The results of our analysis fit the concepts of nonrandom organization of genetic material within and between chromosomes and within the nucleus, represented in terms of chromosome fields, genomic neighborhoods, and expression domains, which are related to the three-dimensional architecture of the eukaryotic genome [83]–[85] and changes in nuclear compartmentalization during transcriptional activation and in the course of evolution [86], [87]. Besides GC content, the organization peculiarities of genomic neighborhoods may be affected by different families of repeated elements, chromosomal position relative to centromere or telomere, and the distance to heterochromatin blocks. Organizational heterogeneity revealed by CS analysis may also be related to this phenomenon. The high correspondence of the CS-heterogeneity scores between the three types of DNA (coding, repetitive, and GDM) permits us to assume that CS-intragenomic heterogeneity may have functional relevance to genome regulation rather than only to reflect different composition and organization of gene-rich and gene-poor regions or local GC enrichment caused by biased gene conversion. Of special interest for such interpretation is that natural GDM sequences display the highest deviation from the corresponding reshuffled sequences, which may hint to a potential role of their organization in the formation of genomic neighborhoods and three-dimensional genome nuclear architecture, hence the regulation of genome dynamics and transcription.

The similarity of effects observed in diverse DNA spaces (coding, repetitive, and GDM) provides evidence of the inter-relatedness of different structural and functional genomic elements. Earlier we showed high positive correlation between interspecific distances based on compositional spectra analysis and corresponding distances based on orthologous genes encoding for information-processing enzymes involved in replication, recombination, DNA repair, and transcription [88]. We hypothesized that high structuring of genome sequences may be associated with intracellular mechanisms where interactions between template DNA and corresponding information processing enzymatic machinery play a leading role. These interactions may derive from several mechanisms: (a) DNA polymerase may have a key role in the evolution of its product (DNA sequence); (b) the structure of repair-recombination enzymes may be evolutionarily more sensitive and “responsive” to changes in its predominant organizational pattern (referred to as “genome dialect”) than proteins involved in structural and metabolic processes; (c) changes in DNA sequences (caused by mutation, recombination, and transposable elements) displayed as changes in genome dialect may improve the mutual correspondence of genome organization and its information processing machinery. These results were obtained on genomes of 20 species of proteobacteria (i.e., with a predominant proportion of protein coding DNA in the genome sequence). The remarkable similarity of the genome heterogeneity patterns across the three considered spaces of vertebrate genomes (coding DNA, repetitive DNA and genome dark matter) found in the current study indicates that co-evolution between information processing enzymes and genome dialect may be a reasonable hypothesis for higher eukaryotes. However, the “genome dialect” concept does not imply unification of organizational variation along the noncoding part of the genome. Indeed, we recently found that compositional and organizational variability patterns in GDM contain sufficient information to reveal conserved parallelism upon a modified dot-plot genome comparison between humans and apes [Kirzhner et al. unpublished results].

The obtained results suggest that the heterogeneity of genomic sequences is a product of a complex interplay between organizational and compositional heterogeneities. The influence of compositional heterogeneity is obvious because any two sequences with diverse nucleotide composition necessarily differ in their compositional spectra. However, according to our results, any two sequences with a very similar composition may still have very different compositional spectra, which probably would be better named “organizational spectra” because they reflect organizational rather than compositional differences of the sequences. Simultaneously, organizational heterogeneity of human genome segments with the same nucleotide composition displays a strong dependence on the deviation of GC content from 50%. Permutation tests indicate that natural genomic sequences do not utilize the whole potential of sequence variation “offered” by their composition: their organizational heterogeneity proved much lower than the heterogeneity of corresponding reshuffled sequences with the same composition. The analysis of inter- and intra-chromosomal heterogeneities forces us to assume the existence of some basic (predominant) organizational patterns in each genome. The degree of deviations of the organizational patterns (genomic states) presented in each chromosome from the predominant patterns determines the level of intra- and, therefore, inter-chromosomal heterogeneities.

We further speculate that in parallel to compositional (GC) isochores, genome sequences deviating from the basic organizational pattern(s) also form a mosaic structure of “organizational” isochores calling for corresponding genome segmentation analysis. An example of such an approach based on the analysis of abundances of tri-nucleotide words was provided by Bingham and co-authors [89]. Chromosomal segments with organizational patterns that are similar in their coding, repetitive and GDM subsequences, may represent the aforementioned neighborhoods involved in spatial organization of the nucleus [87], [90]. A new analysis is underway in our lab aimed at testing for possible association between genome-wise distribution of organizational “isochores” and various evolutionary and functional features, such as rate of gene duplications, indels and SNPs, hotspots of transcription, rate of mutation and recombination, and distribution of linkage disequilibrium blocks.

## References

[1] Karlin S, Ladunga I, Blaisdell BE (1994). Heterogeneity of genomes: measures and values.. Proceedings of the National Academy of Sciences of the USA.

[2] Lercher MJ, Urrutia AO, Pavlícek A, Hurst LD (2003). A unification of mosaic structures in the human genome.. Human Molecular Genetics.

[3] Weir BS, Cardon LR, Anderson AD, Nielsen DM, Hill WG (2005). Measures of human population structure show heterogeneity among genomic regions.. Genome Research.

[4] Schmegner C, Hameister H, Vogel W, Assum G (2007). Isochores and replication time zones: a perfect match.. Cytogenetic and Genome Research.

[5] Sellis D, Provata A, Almirantis Y (2007). Alu and LINE1 distributions in the human chromosomes: evidence of global genomic organization expressed in the form of power laws.. Molecular Biology and Evolution.

[6] Eory L, Halligan DL, Keightley PD (2010). Distributions of selectively constrained sites and deleterious mutation rates in the hominid and murid genomes.. Molecular Biology and Evolution.

[7] Malcom CM, Wyckoff GJ, Lahn BT (2003). Genic mutation rates in mammals: local similarity, chromosomal heterogeneity, and X-versus-autosome disparity.. Molecular Biology and Evolution.

[8] Bernardi G (1989). The isochore organization of the human genome.. Annual Review of Genetics.

[9] Li W (2001). Delineating relative homogeneous G+C domains in DNA sequences.. Gene.

[10] Li W (2002). Are isochore sequences homogeneous?. Gene.

[11] Costantini M, Clay O, Federico C, Saccone S, Auletta F (2007). Human chromosomal bands: nested structure, high-definition map and molecular basis.. Chromosoma.

[12] Costantini M, Bernardi G (2008). Replication timing, chromosomal bands, and isochores.. Proceedings of the National Academy of Sciences of the USA.

[13] Ryba T, Hiratani I, Lu J, Itoh M, Kulik M (2010). Evolutionarily conserved replication timing profiles predict long-range chromatin interactions and distinguish closely related cell types.. Genome Research.

[14] Sémon M, Mouchiroud D, Duret L (2005). Relationship between gene expression and GC-content in mammals: statistical significance and biological relevance.. Human Molecular Genetics.

[15] Versteeg R, van SchaikBDC, van BatenburgMF, Roos M, Monajemi R (2003). The human transcriptome map reveals extremes in gene density, intron length, GC content, and repeat pattern for domains of highly and weakly expressed genes.. Genome Research.

[16] Vinogradov AE (2005). Dualism of gene GC content and CpG pattern in regard to expression in the human genome: magnitude versus breadth.. Trends in Genetics.

[17] Jensen-Seaman MI, Furey TS, Payseur Ba, Lu Y, Roskin KM (2004). Comparative recombination rates in the rat, mouse, and human genomes.. Genome Research.

[18] Schmegner C, Hoegel J, Vogel W, Assum G (2007). The rate, not the spectrum, of base pair substitutions changes at a GC-content transition in the human NF1 gene region: implications for the evolution of the mammalian genome structure.. Genetics.

[19] Melodelima C, Gautier C (2008). The GC-heterogeneity of teleost fishes.. BMC Genomics.

[20] Nekrutenko A, Li W-H (2000). Assessment of compositional heterogeneity within and between eukaryotic genomes.. Genome Research.

[21] Costantini M, Bernardi G (2008). The short-sequence designs of isochores from the human genome.. Proceedings of the National Academy of Sciences of the USA.

[22] Gardiner-Garden M, Frommer M (1987). CpG islands in vertebrate genomes.. Journal of Molecular Biology.

[23] Larsen F, Gundersen G, Lopez R, Prydz H (1992). CpG islands as gene markers in the human genome.. Genomics.

[24] Jabbari K, Bernardi G (1998). CpG doublets, CpG islands and Alu repeats in long human DNA sequences from different isochore families.. Gene.

[25] Vinogradov AE (2003). Isochores and tissue-specificity.. Nucleic Acids Research.

[26] Wu H, Caffo B, Jaffee HA, Irizarry RA, Feinberg AP (2010). Redefining CpG islands using hidden Markov models.. Biostatistics (Oxford, England).

[27] Karlin S, Cardon LR (1994). Computational DNA sequence analysis.. Annual Review of Microbiology.

[28] Nussinov R (1980). Some rules in the ordering of nncleotides in the DNA.. Nucleic Acids Research.

[29] Pietrokovski S, Hirshon J, Trifonov E (1990). Linguistic measure of taxonomic and functional relatedness of nucleotide sequences.. Journal of Biomolecular Structure and Dynamics.

[30] Trifonov EN, Brendel V (1986). Gnomic: A dictionary of genetic codes.

[31] Sims GE, Jun S-R, Wu Ga, Kim S-H (2009). Alignment-free genome comparison with feature frequency profiles (FFP) and optimal resolutions.. Proceedings of the National Academy of Sciences of the USA.

[32] Vinga S, Almeida J (2003). Alignment-free sequence comparison — a review.. Bioinformatics.

[33] Hedges SB (2002). The origin and evolution of model organisms. Nature Reviews.. Genetics.

[34] Rosen G, Garbarine E, Caseiro D, Polikar R, Sokhansanj B (2008). Metagenome fragment classification using n-mer frequency profiles.. Advances in Bioinformatics.

[35] Rosen GL, Reichenberger ER, Rosenfeld AM (2011). NBC: the Naive Bayes Classification tool webserver for taxonomic classification of metagenomic reads.. Bioinformatics.

[36] Csurös M, Noé L, Kucherov G (2007). Reconsidering the significance of genomic word frequencies.. Trends in Genetics: TIG.

[37] Sivaraman K, Seshasayee ASN, Swaminathan K, Muthukumaran G, Pennathur G (2005). Promoter addresses: revelations from oligonucleotide profiling applied to the Escherichia coli genome.. Theoretical Biology & Medical Modelling.

[38] van HeldenJ, André B, Collado-Vides J (1998). Extracting regulatory sites from the upstream region of yeast genes by computational analysis of oligonucleotide frequencies.. Journal of Molecular Biology.

[39] Mariño-Ramírez L, Spouge JL, Kanga GC, Landsman D (2004). Statistical analysis of over-represented words in human promoter sequences.. Nucleic Acids Research.

[40] Healy J, Thomas EE, Schwartz JT, Wigler M (2003). Annotating large genomes with exact word matches.. Genome Research.

[41] Chapus C, Dufraigne C, Edwards S, Giron A, Fertil B (2005). Exploration of phylogenetic data using a global sequence analysis method.. BMC Evolutionary Biology.

[42] Dufraigne C, Fertil B, Lespinats S, Giron A, Deschavanne P (2005). Detection and characterization of horizontal transfers in prokaryotes using genomic signature.. Nucleic Acids Research.

[43] Karlin S (2001). Detecting anomalous gene clusters and pathogenicity islands in diverse bacterial genomes.. Trends in Microbiology.

[44] Li W, Stolovitzky G, Bernaola-Galvan P, Oliver JL (1998). Compositional heterogeneity within, and uniformity between, DNA sequences of yeast chromosomes.. Genome Research.

[45] Kent WJ (2002). BLAT — The BLAST-like alignment tool.. Genome Research.

[46] Höhl M, Ragan MA (2007). Is multiple-sequence alignment required for accurate inference of phylogeny?. Systematic Biology.

[47] Kirzhner V, Paz A, Volkovich Z, Nevo E, Korol A (2007). Different clustering of genomes across life using the A-T-C-G and degenerate R-Y alphabets: early and late signaling on genome evolution?. Journal of Molecular Evolution.

[48] Kirzhner V, Frenkel S, Korol a (2011). Minimal-dot plot: “Old tale in new skin” about sequence comparison.. Information Sciences.

[49] Liao B-Y, Chang Y-J, Ho J-M, Hwang M-J (2004). The UniMarker (UM) method for synteny mapping of large genomes.. Bioinformatics.

[50] Kirzhner V, Korol A, Bolshoy A, Nevo E (2002). Compositional spectrum—revealing patterns for genomic sequence characterization and comparison.. Physica A: Statistical Mechanics and its Applications.

[51] Bejerano G, Pheasant M, Makunin I, Stephen S, Kent WJ (2004). Ultraconserved elements in the human genome.. Science.

[52] Ponting CP, Lunter G (2006). Signatures of adaptive evolution within human non-coding sequence.. Human Molecular Genetics 15 Spec No.

[53] Woolfe A, Elgar G (2008). Organization of conserved elements near key developmental regulators in vertebrate genomes.. Advances in Genetics.

[54] Yamada K, Lim J, Dale JM, Chen H, Shinn P (2003). Empirical analysis of transcriptional activity in the Arabidopsis genome.. Science.

[55] Chen L, Zhao H (2005). Negative correlation between compositional symmetries and local recombination rates.. Bioinformatics.

[56] Bell SJ, Forsdyke DR (1999). Deviation from Chargaff's second parity rule Correlate with direction of transcription.. Journal of Theoretical Biology.

[57] Bell SJ, Forsdyke DR (1999). Accounting units in DNA.. Journal of Theoretical Biology.

[58] Deng B (2007). Mismatch repair error implies Chargaff's Second Parity Rule.. Arxiv preprint.

[59] Kirzhner V, Bolshoy A, Volkovich Z, Korol A, Nevo E (2005). Large-scale genome clustering across life based on a linguistic approach.. Bio Systems.

[60] Touchon M, Nicolay S, Audit B, Brodie of Brodie E-B, Aubenton-Carafa Yd' (2005). Replication-associated strand asymmetries in mammalian genomes: toward detection of replication origins.. Proceedings of the National Academy of Sciences of the USA.

[61] Abe T, Kanaya S, Kinouchi M, Ichiba Y, Kozuki T (2003). Informatics for unveiling hidden genome signatures.. Genome Research.

[62] Azad RK, Rao JS, Li W, Ramaswamy R (2002). Simplifying the mosaic description of DNA sequences.. Physical Review E.

[63] Buschiazzo E, Gemmell NJ (2010). Conservation of human microsatellites across 450 million years of evolution.. Genome Biology and Evolution.

[64] Porceddu A, Camiolo S (2011). Spatial analyses of mono, di and trinucleotide trends in plant genes.. PLoS ONE.

[65] Smith AV, Thomas DJ, Munro HM, Abecasis GR (2005). Sequence features in regions of weak and strong linkage disequilibrium.. Genome Research.

[66] Bernardi G (2000). Isochores and the evolutionary genomics of vertebrates.. Gene.

[67] Duret L, Galtier N (2009). Biased gene conversion and the evolution of mammalian genomic landscapes.. Annual Review of Genomics and Human Genetics.

[68] Katzman S, Capra JA, Haussler D, Pollard KS (2011). GBE Ongoing GC-biased evolution is widespread in the human genome and enriched near recombination hotspots.. Genome.

[69] Costantini M, Cammarano R, Bernardi G (2009). The evolution of isochore patterns in vertebrate genomes.. BMC Genomics.

[70] Costantini M, Filippo MDi, Auletta F, Bernardi G (2007). Isochore pattern and gene distribution in the chicken genome.. Gene.

[71] Dermitzakis ET, Reymond A, Lyle R, Scamuffa N, Ucla C (2002). Numerous potentially functional but non-genic conserved sequences on human chromosome 21.. Nature.

[72] Dermitzakis ET, Reymond A, Antonarakis SE (2005). Conserved non-genic sequences — an unexpected feature of mammalian genomes. Nature Reviews.. Genetics.

[73] McLean C, Bejerano G (2008). Dispensability of mammalian DNA.. Genome Research.

[74] Katzman S, Kern AD, Bejerano G, Fewell G, Fulton L (2007). Human genome ultraconserved elements are ultraselected.. Science.

[75] Carpena P, Bernaola-Galván P, Coronado M, Hackenberg M, Oliver J (2007). Identifying characteristic scales in the human genome.. Physical Review E.

[76] Dunham I, Shimizu N, Roe B, Chissoe S, Hunt A (1999). The DNA sequence of human chromosome 22.. Nature.

[77] Grimwood J, Gordon La, Olsen A, Terry A, Schmutz J (2004). The DNA sequence and biology of human chromosome 19.. Nature.

[78] Hillier LW, Graves Ta, Fulton RS, Fulton La, Pepin KH (2005). Generation and annotation of the DNA sequences of human chromosomes 2 and 4.. Nature.

[79] Hattori M, Fujiyama A, Taylor T, Watanabe H, Yada T (2000). The DNA sequence of human chromosome 21.. American Journal of Ophthalmology.

[80] Zody MC, Garber M, Adams DJ, Sharpe T, Harrow J (2006). DNA sequence of human chromosome 17 and analysis of rearrangement in the human lineage.. Nature.

[81] Nusbaum C, Zody MC, Borowsky ML, Kamal M, Kodira CD (2005). DNA sequence and analysis of human chromosome 18.. Nature.

[82] Jurka J, Kohany O, Pavlicek A, Kapitonov VV, Jurka MV (2004). Duplication, coclustering, and selection of human Alu retrotransposons.. Proceedings of the National Academy of Sciences of the USA.

[83] Caron H, van SchaikB, van der MeeM, Baas F, Riggins G (2001). The human transcriptome map: clustering of highly expressed genes in chromosomal domains.. Science.

[84] Cremer T, Cremer C (2001). Chromosome territories, nuclear architecture and gene regulation in mammalian cells. Nature Reviews.. Genetics.

[85] Meaburn KJ (2007). Chromosome territories.. Nature.

[86] Chubb JR, Bickmore WA (2003). Compartmentalization in the light of nuclear dynamics.. Cell.

[87] De S, Babu MM (2010). Genomic neighbourhood and the regulation of gene expression.. Current Opinion in Cell Biology.

[88] Paz A, Kirzhner V, Nevo E, Korol A (2006). Coevolution of DNA-interacting proteins and genome “dialect”.. Molecular Biology and Evolution.

[89] Bingham E, Gionis A, Haiminen N, Hiisila H, Mannila H (2006). Segmentation and dimensionality reduction..

[90] Lieberman-Aiden E, van BerkumNL, Williams L, Imakaev M, Ragoczy T (2009). Comprehensive mapping of long-range interactions reveals folding principles of the human genome.. Science.

